# Environmental concern-based site screening of carbon dioxide geological storage in China

**DOI:** 10.1038/s41598-017-07881-7

**Published:** 2017-08-08

**Authors:** Bofeng Cai, Qi Li, Guizhen Liu, Lancui Liu, Taotao Jin, Hui Shi

**Affiliations:** 1 Center for Climate and Environmental Policy, Chinese Academy for Environmental Planning, Beijing, 100012 China; 2 State Key Laboratory of Geomechanics and Geotechnical Engineering, Institute of Rock and Soil Mechanics, Chinese Academy of Sciences, Wuhan, 430071 China; 3 University of Chinese Academy of Sciences, Beijing, 100049 China

## Abstract

Environmental impacts and risks related to carbon dioxide (CO_2_) capture and storage (CCS) projects may have direct effects on the decision-making process during CCS site selection. This paper proposes a novel method of environmental optimization for CCS site selection using China’s ecological red line approach. Moreover, this paper established a GIS based spatial analysis model of environmental optimization during CCS site selection by a large database. The comprehensive data coverage of environmental elements and fine 1 km spatial resolution were used in the database. The quartile method was used for value assignment for specific indicators including the prohibited index and restricted index. The screening results show that areas classified as having high environmental suitability (classes III and IV) in China account for 620,800 km^2^ and 156,600 km^2^, respectively, and are mainly distributed in Inner Mongolia, Qinghai and Xinjiang. The environmental suitability class IV areas of Bayingol Mongolian Autonomous Prefecture, Hotan Prefecture, Aksu Prefecture, Hulunbuir, Xilingol League and other prefecture-level regions not only cover large land areas, but also form a continuous area in the three provincial-level administrative units. This study may benefit the national macro-strategic deployment and implementation of CCS spatial layout and environmental management in China.

## Introduction

Carbon dioxide (CO_2_) Capture and Storage (CCS) technology will not only play an important role in reducing CO_2_ emissions from the combustion of fossil fuels, but will also be an important option for significantly reducing direct emissions of CO_2_ during the production processes of many industries^1–7^. Therefore, CCS will have a significant meaning related to low carbon development and new urbanization processes in China^8–12^. In China, the term CO_2_ Capture, Utilization and Storage (CCUS) is much more popular than CCS because of its focus on using and storing CO_2_^13–17^. In the past decade, CCS technology has experienced a rapid development, which had been proved to be a feasible and widely used technology worldwide. As an emerging new technology that can be used to combat climate change, CCS faces serious challenges such as uncertainties of reservoir properties^18–24^, high energy consumption, high investment and uncertainties of environmental risks during the process of implementation^25–27^. Particularly, the geological complexity of CCS creates uncertain environmental impacts; environmental risks restrict awareness and acceptance of CCS by government agencies and the public for this type of effective CO_2_ mitigation technology^28–30^.

The environmental risks of CCS mainly include environmental damage caused by CO_2_ leakage from geological storage sites. The prominent environmental risk is associated with potential CO_2_ leakage^31, 32^. CO_2_ leaks from geological storage reservoirs mainly occur when sequestered CO_2_ moves up to near the surface from deep subsurface, which may affect the local environment^33^. When CO_2_ is injected into underground reservoirs, the CO_2_ may leak through the following patterns: leaks through pores of low-permeability caprock (such as shale or mudstone); laterally leaks through unconformity (a buried erosional surface located between two formations or strata with different ages, indicating that sediment deposition was not continuous) or leakage through pores in rock; leaks through fractures in the caprock, ruptures or geological faults; leaks through wells or abandoned wells, including leakage between the cement and the outside of the casing, between the cement and the inside of the metal casing, within the cement plug itself, through deterioration (corrosion) of the metal casing, deterioration of the cement in the annulus and leakage in the annular region between the formation and the cement^34, 35^. The potential leakage pathways mentioned above and resulting environmental risks should be analyzed and evaluated in the pre-feasibility study of CCS sites^36^.

A proper site selection of CCS projects can effectively avoid potential environmental risks and improve the acceptance of those projects by government authorities and the public. Moreover, site selection is a time-consuming and daunting task^37–39^. In general, the site selection of a CCS project typically includes 2–3 phases: screening, preliminary site selection and preliminary description of the target site^40–42^. The phases used in analyzing site selection may differ slightly around the world, but the baseline content is similar^43–46^. The first step in site selection involves screening of suitable regions against specific suitability criteria along with a more or less parallel assessment of storage capacity^47–49^. The site selection of a CCS project described here includes screening at either the national level or a sedimentary basin level, which focuses on a screening framework of environmental indicator construction and regional environmental analysis. This stage does not consider source-sink matching. Bachu^1^ proposed a systematic approach for the assessment and selection of methods and sites for CO_2_ sequestration in geological media. The basin selection criteria could be classified into geological, hydrodynamic and geothermal, hydrocarbon potential and basin maturity, economic and political and societal categories. Then, a roadmap for site selection using the transform of the geological space into the CO_2_ phase space was proposed based on a geoscience based analysis that includes suitability, inventory, safety and capacity^50^. In addition, a set of 15 criteria with several classes was developed for the assessment and ranking of sedimentary basins in terms of their suitability for CO_2_ sequestration. A basin’s individual scores are summed to a total score using weights that express the relative importance of different criteria using a parametric normalization procedure^37^. This screening methodology has been widely adopted and modified by researchers worldwide^51–54^, and this method has been successfully applied in some cases in China^45, 55^. Damen, *et al*.^56^ performed a study to identify potential worldwide opportunities for the early application of CO_2_ sequestration by using a Geographical Information System (GIS) to combine worldwide CO_2_ point sources with oil and coal fields. They defined an early opportunity as a situation including high-purity CO_2_ point source that provides CO_2_ at low costs to oil or coal fields with the goal of enhancing oil production or coal bed methane production, at a site where CO_2_ is simultaneously sequestered. Li, *et al*.^57^ ranked the aquifer storage sites in Japan in terms of potential CO_2_ storage capacity and potential CO_2_ supply, both of which significantly affect the storage economics. Meyer, *et al*.^58^ reported on regional screening, site selection and geological characterization of a potential storage site in northeastern Germany while considering the capacity limit for pipeline transport distance of up to about 300 km based on economic reasons. Oldenburg^38^ developed a screening and ranking framework for CO_2_ geological storage on based on health, safety, and environmental (HSE) risk arising from CO_2_ leakage. That framework did not explicitly consider the environmental concerns such as national parks and human distribution. Li, *et al*.^59^ applied a slightly revised screening and ranking framework based on HSE risk that was developed by Oldenburg^38^ to evaluate the risk of leakage for China’s first full-chain Shenhua CCS demonstration site in the Ordos Basin. Grataloup, *et al*.^60^ proposed a site selection method with different objectives, such as storage optimization and risk minimization, with respect to regulations and spatial constraints, gave full consideration to the social and economic aspects of CCS. The corresponding criteria were classified into “killer criteria” and “site-qualification criteria”. This multi-criteria method was applied on the PICORE study area by a GIS tool. The GIS tool was also used in the METSTOR project in France to look for potential CO_2_ storage zones based on an interactive map of the CO_2_ storage capacities of various sites^61^. Mathias, *et al*.^47^ presents a simple method for estimating pressure buildup caused by the injection of supercritical CO_2_ into a saline formation, and the limiting pressure of fracking in the target formations. Such a method will be useful for screening and selecting sites for CO_2_ sequestration with the goal of identifying sites that are worthy of further investigation. Ramírez, *et al*.^53^ used the main aspects of multi-criteria analysis in a linear aggregation tool as a method used to screen and rank off- and on-shore reservoirs suitable for long-term large scale CO_2_ storage in the Netherlands. The screening of storage options was based on a set of three criteria, i.e. potential storage capacity, storage costs and the amount of effort needed to manage the risk involved. Hsu, *et al*.^62^ proposed an analytic network process approach for the selection of potential sites for CO_2_ geological storage as a basis for further geological feature exploration and the simulation of transport characteristics. A multi-criteria decision model with eight evaluation criteria was proposed with the consideration of site selection as a complex multi-criteria decision-making problem. Raza, *et al*.^63^ presented a general criterion based on local-scale projects under the consideration of storage capacity, injectivity, trapping mechanisms, containment and cost. A group of key parameters including reservoir and well types, classes of minerals, residual gas and water saturations, subsurface conditions, rock types, wettability, properties of CO_2_, and sealing potentials were analyzed to provide an insight into the suitable selection of storage sites.

It is important to clarify that the aforementioned research on CCS site screening did not fully consider environmental constraints, but was more focused on the potential for underground sequestration and related economic factors. Wei, *et al*.^64^ developed a preliminary sub-basin scale evaluation framework of site suitability for onshore aquifer-based CO_2_ storage in China based on a multi-criteria analysis framework, which considers four objectives: storage optimization, risk minimization and storage security, environmental restrictions regarding surface and subsurface use and economic considerations. That study used GIS-based evaluation tool to conduct the application of the framework. In their research, only three environmental restrictions regarding existing surface and subsurface use were considered. These included the proximity of cities, the distribution of natural resources and coal resources as well as the distance from the CCS site to existing deep coal mines. That study also implemented a screening framework to reflect potential damage or economic impacts to the geological or terrestrial environment. The government of China attaches great importance to environmental impacts and risks related to CCS technology. On 20^th^ June 2016, the Ministry of Environmental Protection of the People’s Republic of China issued *Technical Guidelines on Environmental Risk Assessment for Carbon Dioxide Capture*, *Utilization and Storage* (*on Trial*)^65^. The authors of this paper were major participants in the formulation of these guidelines. While considering the supervision of the government of China of environmental impacts and risks of a CCS project, the present paper first proposes an environmental optimization method for CCS site selection that mirrors the method of delineation of China’s ecological red line. In addition, this paper establishes a GIS spatial analysis model of environmental optimization for CCS site selection, and carries out a detailed analysis of CCS site selection in China based on environmental optimization.

The ecological red line system (ERLS), initiated in 2013, provides one of the important set of environmental management standards and guidelines in China and designates areas to be protected from human activities^66, 67^. The ERLS has been stressed by the Communist Party of China (CPC) in the third Plenary Session of the 18^th^ CPC Central Committee, and marks a great breakthrough in environment protection in China. In 2015, the Ministry of Environmental Protection of the People’s Republic of China promulgated a *Technical Guideline for Ecological Protection Red Line Delineation* ([2015] No. 56) to improve the implementation and delineation methods of ecological red line designation^66^. The ERLS plays a very important role in the protection of ecological resources, ecologically fragile zones, and biodiversity in China^68^. The essence of the ERLS is to spatially divide the human land management and activities into different regions, so as to achieve a desirable level of ecological protection for ecosystems. By combining bottom-up with top-down management characteristics in China, the ERLS continues to play an increasingly important role in environment management in China.

Enlightened by the methods and procedures provided in the ERLS, this paper established a spatial model to identify and evaluate the degree of suitability for the implementation of CCS projects in different regions of China, so as to provide a reference for the government’s policy-making and regional layout of CCS projects. Below, Section 2 describes the method and data used for site-specific screening. Section 3 shows the results of the assessment and analysis while Sections 4 provides policy proposals and a discussion of the findings.

## Methods and Data

### Environmental elements and indices

CO_2_ leakage from a human-created storage location may be harmful to human health and create negative effects to ecosystems, soils, and groundwater, etc. (Table 1). The leaked CO_2_ dissolved in the groundwater could induce the motivation of some toxic metal element^69, 70^. There may also be acidification of soils and displacement of oxygen in soils. Additionally, if leakage to the atmosphere, CO_2_ may lead to a suffocation of humans or animals, or effect on plants above ground^71^.

**Table 1:** Main environmental elements impacted by CO_2_ geological storage.

Environmental elements	Negative impact	Fatal or serious impact
Groundwater and Surface Water	Low acidity would be caused at 0.2~2% concentration of CO_2_, but it will not make a significant difference; If CO_2_ concentration >2%, it will cause moderate acidity and corrosion.	If CO_2_ concentration >6%, it will lead to an increased acidity, enhanced corrosion and loss of irrigation effect.
Vegetation	If CO_2_ concentration >5%, it will have a detrimental effect on plant health and yield; Concentration of 5–30% will have a serious impact.	If CO_2_ in the soil gas exceeds over 20% in the long term (weeks or months), it will lead to dead zones, and no naked eye visible plants survived. More than 30% are considered fatal levels of plant life.
Human Health	1–3% CO_2_ concentration will cause shortness of breath, headaches and sweating, which will appear a physiological adaptation without negative effects; 3–5% CO_2_ concentration will cause shortness of breath, high blood pressure and some discomfort. CO_2_ concentration >5% will lead to physical and mental harm, and loss of consciousness.	CO_2_ concentration >10% would lead to severe symptoms, including rapid loss of consciousness, coma or death may. If exposure to such an environment in a long time, CO_2_ concentration exceed 25–30% will lead to loss of consciousness or even lead to respiratory arrest and death.

**Figure 1:**
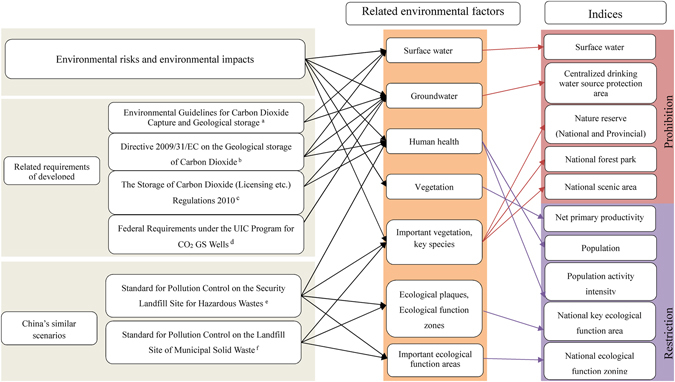
Factors and indices related to the environmental risks and effects of CO_2_ capture and storage. Note for Fig. 1: ^a^The Australian Environment Protection and Heritage Committee, *Environmental Guidelines for Carbon Dioxide Capture and Geological storage —* 2009, claims: all CCS projects must subject to environmental assessment during the legislative phase. All environmental risk assessment must contain an assessment of groundwater resources so as to protect regional water resources^88^. ^b^European Union, *Directive* 2009/31/*EC of the European Parliament and of the Council on the Geological Storage of Carbon Dioxide*, claims: only areas with no significant risks of leakage and with no significant environmental and health risks can be selected as sequestration sites, and water resources or water protection areas should not be used for carbon dioxide sequestration^89^. ^c^United Kingdom, *The Storage of Carbon Dioxide* (*Licensing etc*.) *Regulations* 2010, claims: licenses should not be issued for CO_2_ sequestration in water source areas; a sequestration license may be issued on the condition that the proposed sequestration site is free of leakage, environmental hazards and human health risks (http://www.legislation.gov.uk/uksi/2010/2221/pdfs/uksi_20102221_en.pdf). ^d^US Environmental Protection Agency, *Federal Requirements Under the Underground Injection Control* (*UIC*) *Program for Carbon Dioxide* (*CO*_2_) *Geologic Sequestration* (*GS*) *Wells*. The bill took effective on 10^th^ January 2011 for geological storage wells of CO_2_. The purpose of this bill is to establish a new class, namely Class VI, to protect underground drinking water resources^90^. ^e^ *Standard for Pollution Control on the Security Landfill Site for Hazardous Wastes* (*GB 18598-2001*/*XG1-2013*)(*China*) claims^74^: site locations and distances from hazardous waste landfills to the surrounding population should be determined based on the conclusion of an environmental impact assessment, which should be identified and approved by the administrative department of environmental protection. The administrative approval will be treated as a basis for planning control. Landfill sites should not be selected in the area of urban planning and agricultural development, agricultural protection areas, nature reserves, scenic spots, heritage/archaeology conservation areas, protection areas for drinking water resources, long term planning areas for water supply, mineral resources districts and the other areas particularly in need of protection. ^f^The *Standard for Pollution Control on the Landfill Site of Municipal Solid Waste* (*GB 16889-2008*) (*China*) claims^75^: landfill sites should be consistent with regional environmental planning, environmental health facilities planning and local urban planning. Landfill sites should not be selected in areas of urban planning and agricultural development, agricultural protection areas, nature reserves, scenic spots, heritage/archaeology conservation areas, protection areas of drinking water resources, long term planning area for water supply, mineral resources districts, military sites, state secret areas and other zones that need special protection. The site selection of landfills should avoid wetlands.

The environmental effects and risks of CCS have caused the United States, European Union, Australia and other developed countries to formulate environmental regulations and provisions to protect ecosystems from the national level, including water sources, groundwater and human health, etc. The Ministry of Environmental Protection of the People’s Republic of China promulgated the *Technical Guidelines on Environmental Risk Assessment for Carbon Dioxide Capture*, *Utilization and Storage*, which categorized risk receptors in the CCS environmental assessment as the human population, animals and plants and other life forms and closely related groundwater, surface water, air, soil and other environmental media^65, 72, 73^. In addition, China has been implementing similar regulations which could be referenced by the site selection criteria of CO_2_ geological storage, such as the *Standards for Pollution Control on the Security Landfill Site for Hazardous Wastes* (*GB 18598-*2*001*/*XG1-*2*013*)^74^ and the *Standards for Pollution Control on the Landfill Site of Municipal Solid Waste* (*GB 16889-*2*008*)^75^, etc. The above standards proposed restriction and prohibition requirements for site selection. Considering standards and guidelines of environmental elements (Fig. 1) mentioned above, this paper identified water resource (groundwater and surface water), vegetation and human health as the major environmental elements affected by the environmental risks and environmental impacts of CO_2_ geological storage. Compared with these elements, other elements are less affected (Table 1). Therefore, during optimization of the environmental impact for the site selection process for CO_2_ geological storage sites, it is important to consider the characteristics and vulnerability of water resources, vegetation, wildlife, and human health in target areas of CCS projects. In addition, China has strategically implemented sustainable development plans related to main function zoning and ecological function zoning, which forms an important macro guidance plan for China’s economic development and environmental protection. The environmental risks of CO_2_ geological storage require that site selection must meet the requirements of the state’s macro spatial planning, i.e. China’s main function zoning and the national ecological function division planning. Table 2 describes main environmental elements affected by CO_2_ geological storage, and elaborates the characterization of indices for environmental elements.

**Table 2:** Evaluation index system for environmental risks and environmental impacts of CO_2_ geological storage.

Class	Content	Environment elements	Description
Prohibited index	Surface water	Surface water	Rivers and lakes.
	Protection area of centralized drinking water resources	Groundwater	According to national centralized drinking water resources, the maximum radius (10000 m) of the second protected areas was adopted, which listed in *Technical Guideline for Delineating Source Water Protection Areas* (HJ/T338-2007), as groundwater resources protection area in this research.
	Nature reserve (national and provincial)	Important vegetation, key species	All lands, land waters and sea areas where representative natural ecosystems, the naturally concentrated distribution of rare and endangered native wildlife species and nature relics with special meaning and other protective objects located on, are required to set aside special area for protection and management according to relevant law and regulations.
	National forest park	Important vegetation, key species	Forest landscape with beautiful scenery, the area with concentrated humanities, high ornamental value, high scientific value, and high cultural values, special geographical site, regional representation, sound tourism service facilities, high visibility, and the area convenient for people to visit, rest or carry out scientific, cultural and educational activities.
	National park of China	Important vegetation, key species	Natural landscape and human landscape can reflect important natural change process and major historical and cultural development process. The area basically keeps in a natural state or historical original appearance with national representative characteristics.
Restricted index	National key ecological function areas (national main function zoning)	Ecological patch, ecological function areas	The national main function zoning is a strategic planning, space layout and binding plan for China’s national territorial spatial development. This plan puts forward clear requirements in different economic and social activities for China. The national key ecological function areas are important regions for maintaining regional ecological security pattern, biodiversity, realizing a virtuous circle and sustainable utilization of wildlife resources, protecting the natural ecological system and habitat of important species.
	National ecological function zoning	Important ecological function areas	National ecological function zoning is based on regional ecosystem, ecological sensitivity and ecological service function of spatial distribution pattern, and divided area into different ecological function regions. National ecological function zoning has defined the concept of national important ecological function areas which plays an important role in guiding the site selection of CO_2_ geological storage. The two areas of water conservation and biodiversity conservation in national key ecological function zoning are most sensitive relative to geological storage project.
	Net Primary Productivity (NPP)	Land vegetation	NPP represents the active degree of land vegetation.
	Population	Human health	To use LandScan data (http://web.ornl.gov/sci/landscan/landscan_data_avail.shtml). LandScan global population dataset was developed by U.S. Department of Energy’s Oak Ridge National Laboratory (ORNL), which is the most accurate and reliable around the world. It has a distribution model and the best resolution of global dynamic statistical analysis. LandScan population data is an accepted standard by U.S. Department of Defense and the State Department to assess population risk.
	Population activity intensity	Human health	To use big data with spatial information from Weibo Blog. 1 km grid spatial distribution of population data is assumed that people basically do not move, so the affected population can be assessed based on specific population density. However, human movement and activity capacity are very strong, even if their residences (based on demographic and census) are fixed. It is likely that most of the time they are engaged in activities in other area.

### Evaluation model

**Figure 2:**
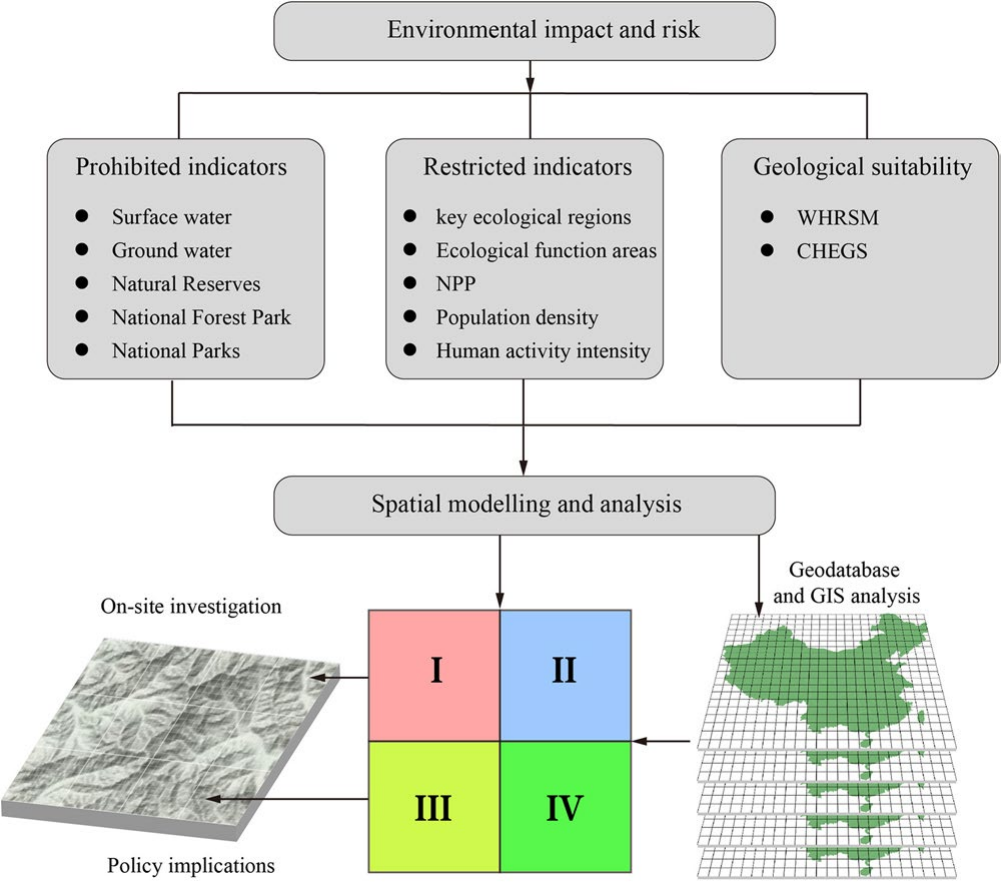
Schematic diagram for environment optimization based site selection for CO_2_ geological storage. Note: WHRSM, Institute of Rock and Soil Mechanics, Chinese Academy of Sciences; CHEGS, China Geological Survey Center for Hydrogeology and Environmental Geology Survey. Four categories of suitability for CO_2_ geological storage include I and II, III and IV with increasing suitability. The map in this figure were generated by software ArcGIS 9.2 (http://www.esri.com/arcgis/about-arcgis) based on our own data. The China spatial boundary GIS data is from the National Geomatics Center of China (affiliated with National Administration of Surveying, Mapping and Geoinformation of China), which provides the basic GIS data for China.

**Figure 3:**
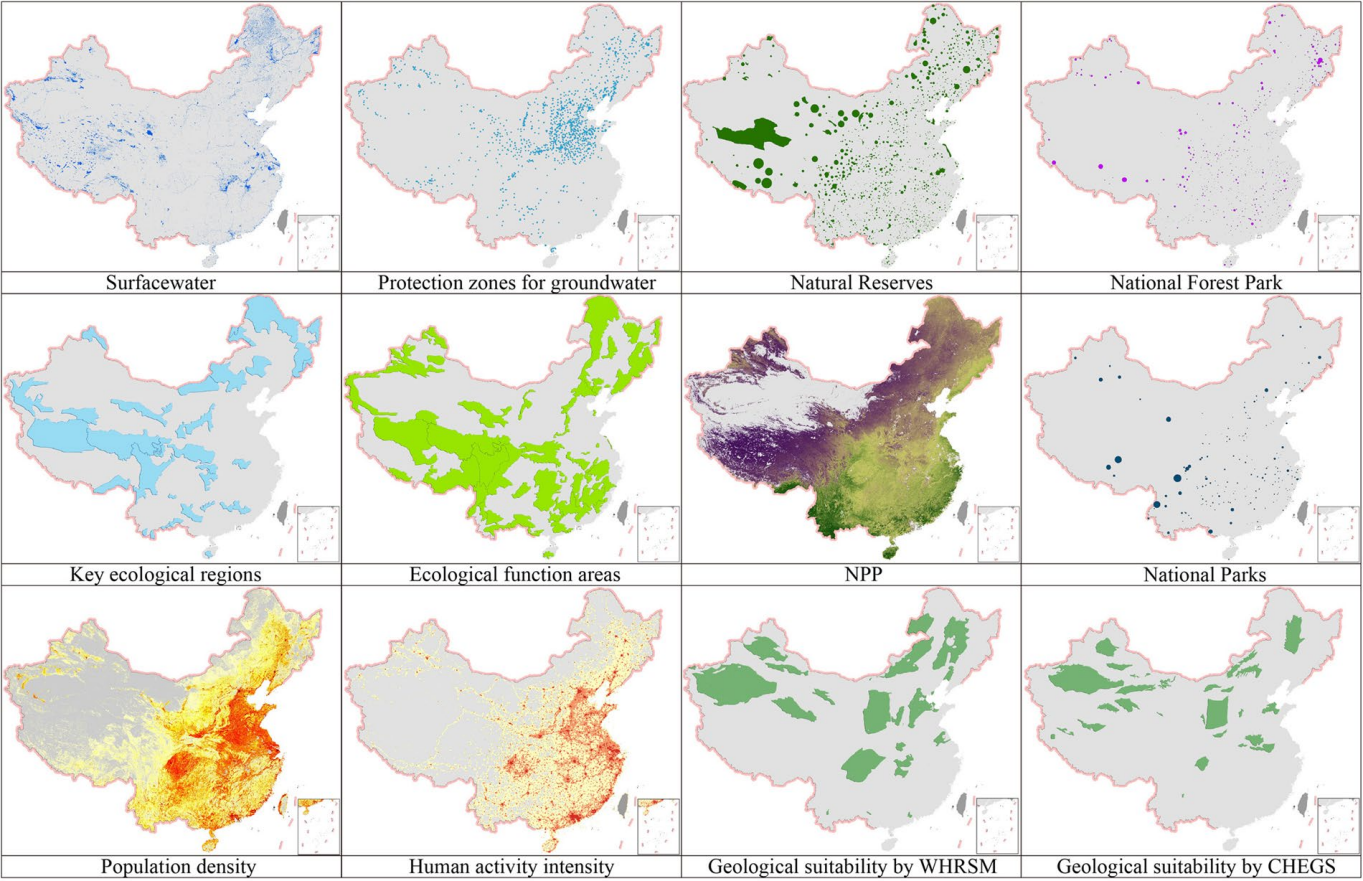
Dataset for basic environmental elements (Sources: Wuhan Institute of Rock and Soil Mechanics, Chinese Academy of Sciences (WHRSM); China Geological Survey Center for Hydrogeology and Environmental Geology Survey, Baoding, China (CHEGS)). Note: NPP, net primary production. The map in this figure were generated by software ArcGIS 9.2 (http://www.esri.com/arcgis/about-arcgis) based on our own data. The China spatial boundary GIS data is from the National Geomatics Center of China (affiliated with National Administration of Surveying, Mapping and Geoinformation of China), which provides the basic GIS data for China.

**Figure 4:**
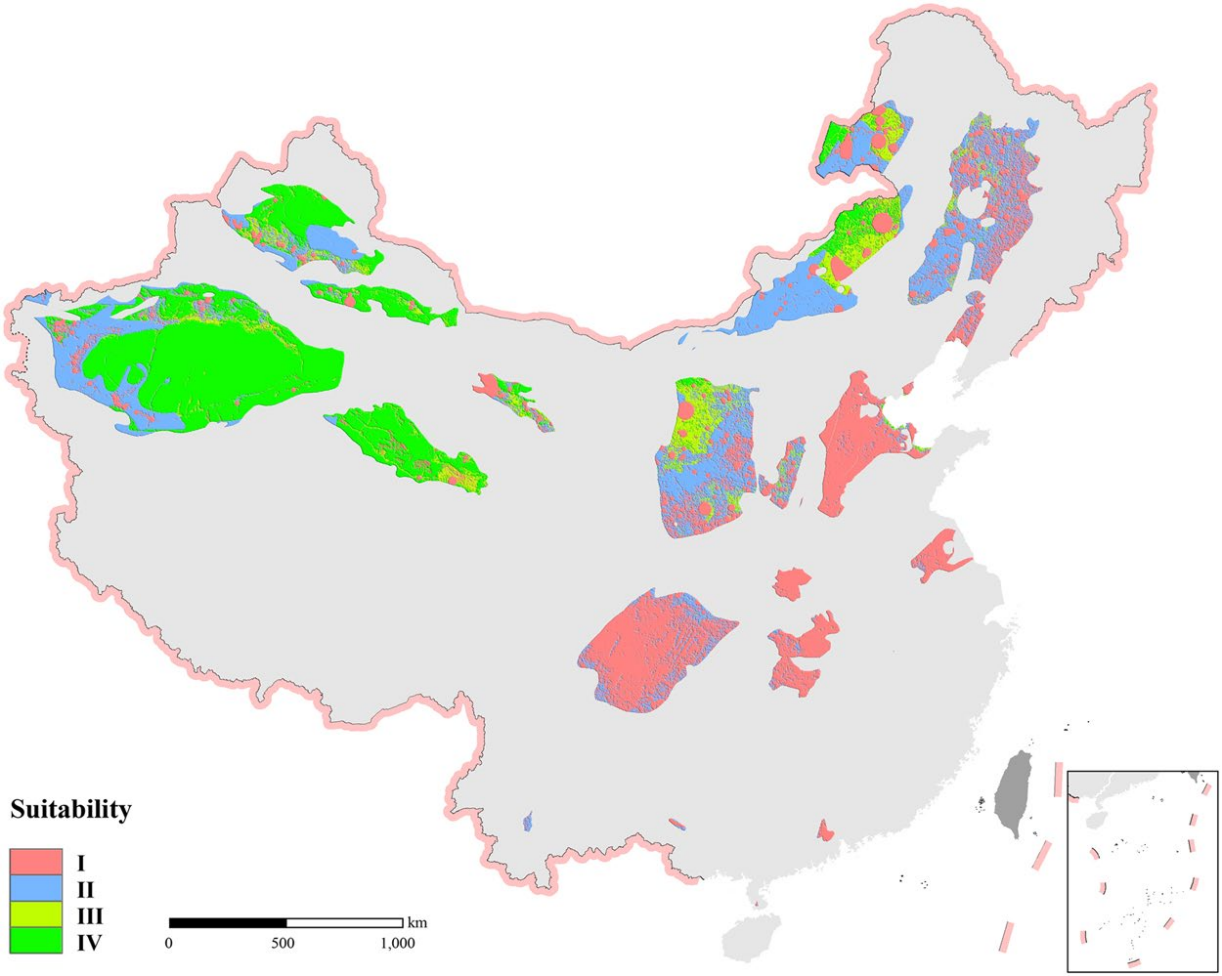
Environmental suitability mapping related to CO_2_ capture and storage site selection in China. Note: Four categories of suitability for CO_2_ geological storage include I (prohibited/unsuitable) and II, III and IV with increasing suitability. The map in this figure were generated by software ArcGIS 9.2 (http://www.esri.com/arcgis/about-arcgis) based on our own data. The China spatial boundary GIS data is from the National Geomatics Center of China (affiliated with National Administration of Surveying, Mapping and Geoinformation of China), which provides the basic GIS data for China.

**Figure 9:**
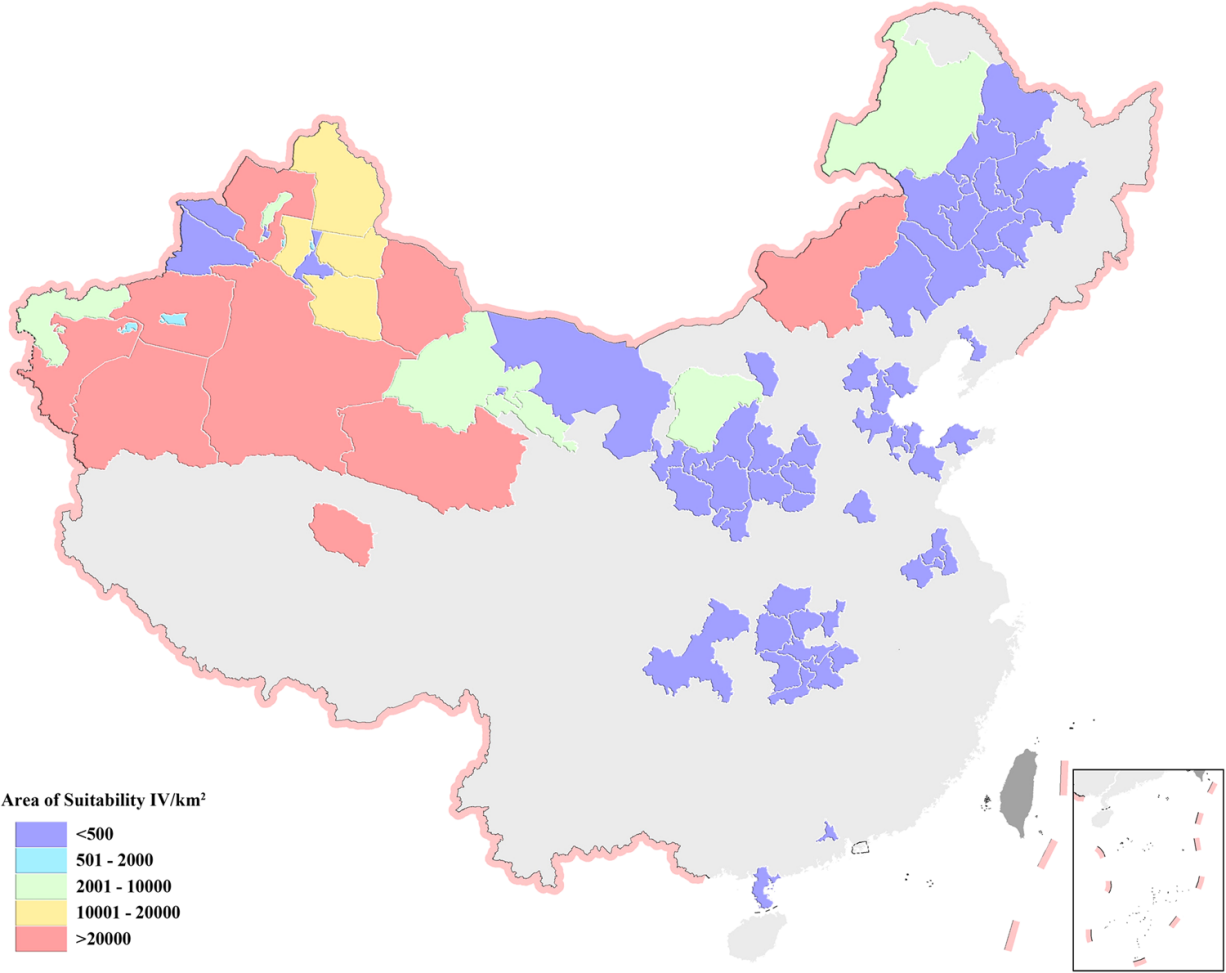
Mapping of areas classified as environmentally suitable for CO_2_ capture and storage as class IV in China’s prefecture-level regions. Four categories of suitability for CO_2_ geological storage include I and II, III and IV with increasing suitability. The map in this figure were generated by software ArcGIS 9.2 (http://www.esri.com/arcgis/about-arcgis) based on our own data. The China spatial boundary GIS data is from the National Geomatics Center of China (affiliated with National Administration of Surveying, Mapping and Geoinformation of China), which provides the basic GIS data for China.

**Figure 10:**
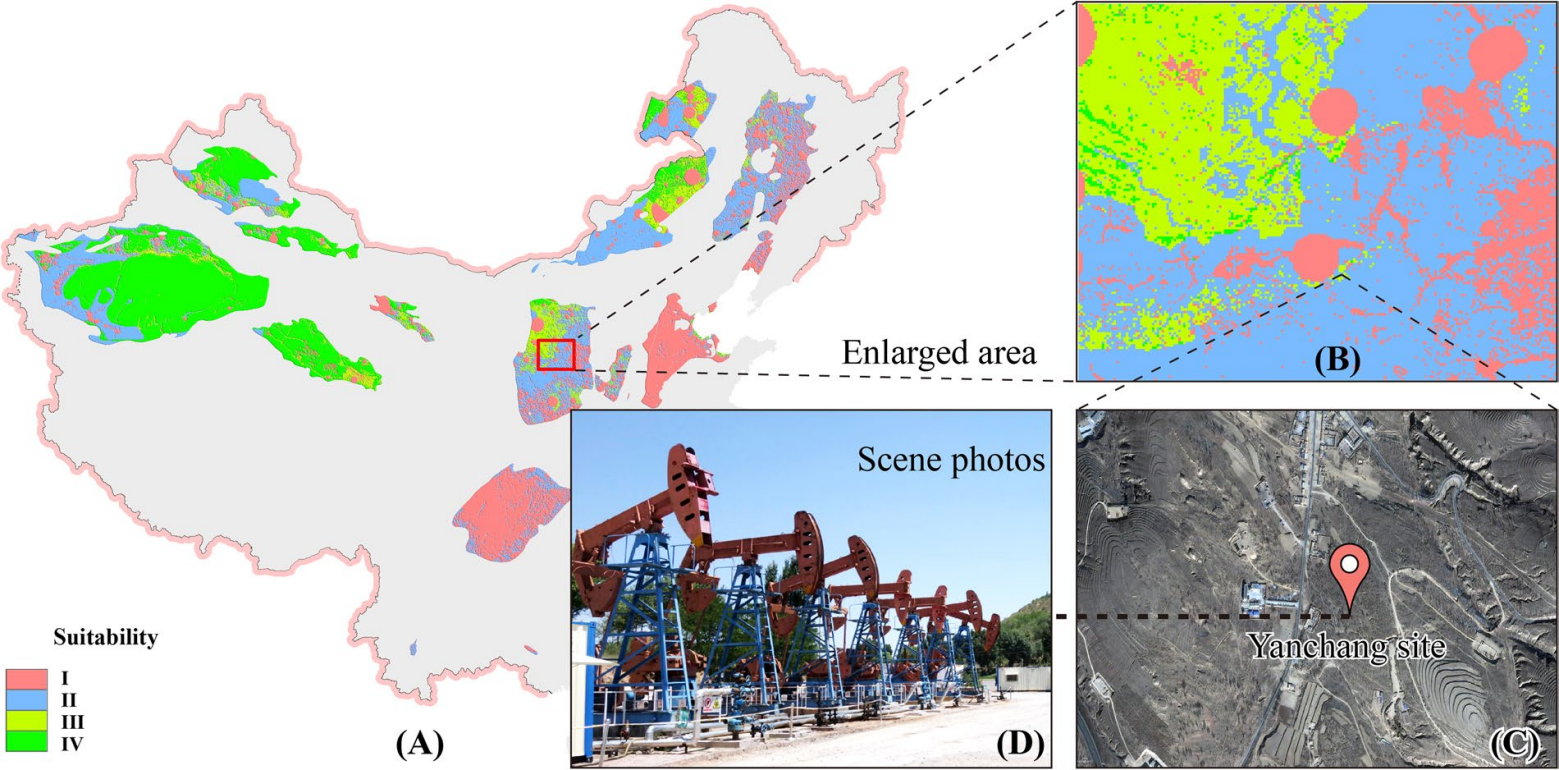
Environmental suitability of the Yanchang CO_2_ Capture, Utilization and Storage project. Four categories of suitability for CO_2_ storage include I and II, III and IV with increasing suitability. (**A**) Environmental suitability results of China; (**B**): Enlarged area in (**A**) of Yanchang site; (**C**): Remote sensing images of Yanchang site; (**D**): Scene photo of Yanchang site. The map in this figure were generated by software ArcGIS 9.2 (http://www.esri.com/arcgis/about-arcgis) based on our own data. The China spatial boundary GIS data is from the National Geomatics Center of China (affiliated with National Administration of Surveying, Mapping and Geoinformation of China), which provides the basic GIS data for China. The satellite map in Fig. 10C was obtained from a commercial source image bought under a contract from the Institute of Remote Sensing and Digital Earth, Chinese Academy of Sciences.

Based on the methods and techniques in the *Technical Guideline for Ecological Protection Red Line Delineation* ([2015] No. 56), multi-factor analysis and GIS spatial analysis, this paper attempts to determine how to build a spatial evaluation model for CCS site selection with a focus of environment optimization (Fig. 2). The ERLS has a good solution for weight assignments for different indicators, without considering duplication between the indicators. This method defines the importance of each evaluation region based on the most important environmental elements in the region. The quartile method is used for value assignment for specific indicators. This type of statistical analysis is used to describe the data, especially the degree of dispersion for skewed data. The data are arranged in ascending order and then are divided into four equal parts so that each part of the data volume account for 25% of entire data volume. The first quartile, also known as the “lower quartile,” is equal to the smallest 25% of all data. The second and third quartiles, also known as “median”, are equal to 50% data of all data. The fourth quartile, also known as “upper quartile”, equal to the largest 75% of all data. A suitable CCS site may be selected based on the spatial model. Meanwhile, various types of information are analyzed based on the site suitability and characteristics while using case studies to verify the suitability of an area. The spatial boundary of suitable CCS sites and related detailed records will ultimately be created. The GIS spatial analysis model used here allows a thorough analysis of the identification of optimal environments for CO_2_ geological storage, including the projection and conversion of the original data, buffer analysis using point distribution data, conversion of data from vector to raster format, re-sampling of raster data, quartile classification operations by ArcMap Quantile functions, and spatial calculations. The maps in Figs 2–4, 9, 10 were generated by software ArcGIS 9.2 based on our own data. The China spatial boundary GIS data is from the National Geomatics Center of China (affiliated with National Administration of Surveying, Mapping and Geoinformation of China), which provides the basic GIS data for China.

### Data

The basic data required for the model employed in this paper included two parts: suitability evaluation results of CO_2_ geological storage in sedimentary basins and basic data related to optimal site selection based on environmental issues in China (Table 3 and Fig. 3). Sedimentary basins are considered as suitable sites for CO_2_ geological storage for the stable structure with the large capacity and well injectivety. Some pilot projects have been constructed and operated in the sedimentary basins, such as Shenhua CCS project in Ordos basin. Additionally, lots of oil and gas fields distrusted in basins provide potential sites for CO_2_-EOR, CO_2_-EGS, or CO_2_ storage in depleted oil or gas fields. For example, the Songliao basin has been proved to have large storage capacity in deep saline aquifers with good reservoir-caprock properties^76^, moreover, several CO_2_-EOR experiments have been carried out in the basin since the 1960, and the theoretical storage capacity of CO_2_ in the oilfields of the Basin is large to 2.36 × 10^9^t^77^.

**Table 3:** Basic data sheet.

Index	Content	Data sources	Processing method	Suitability class			
				IV	III	II	I
Prohibited index	Surface water	Globeland30-2010, 30 m spatial resolution	/	/			In Scope of surface water distribution
	Protection area of centralized drinking water resources	Environmental planning basic database of CAEP		/			In Scope of protection area of centralized drinking water resources
	Nature reserve (national and provincial)	MEP China^91^		/			In scope of nature reserve, national forest park and national park of China
	National forest park	State Forestry Administration^92^		/			
	National park of China	2015 directory of national 5 A scenic spots		/			
Restricted index	National main function zoning	State Council^93^	/	/	/	Within the boundary of key ecological function areas	/
	National ecological function zoning	MEP China^94^	/	/	/	Within the boundary of important ecological function areas	/
	Net Primary Productivity (NPP)	To calculate from synthetic NDVI data products and reflectivity data by using CASA model combined with MODIS (250 meters every 16 days)	Quartile method (Sort data in ascending)	0~25% Area	25~50% Area	50~75% Area	75~100% Area
	Population	LandScan data					
	Population activity intensity	Data acquisition from Sina Weibo uses its official API to get APP Key, APP Secret, and the authorized user access token.					

Note: Suitability class I covers prohibited indexes for CCS site selection, i.e. it is prohibited to construct CCS projects in areas with this suitability class; Suitability classes II, III and IV indicate environmental suitability of CCS site selection successively increased.

We synthetically considered the two evaluation results of CO_2_ geological storage suitability, issued by the Wuhan Institute of Rock and Soil Mechanics, Chinese Academy of Sciences (WHRSM) and the China Geological Survey Center for Hydrogeology and Environmental Geology Survey (CHEGS). Both results were very similar (Fig. 3) under the same assumption used for the analytic hierarchy process assessment. However, the conclusion of WHRSM was more sophisticated in detail. Through our integrated assessment and consideration, we tend to accept WHRSM assessment results. WHRSM applied four indices, including seismic intensity for crust stability, terrestrial heat flow for geothermal geological conditions of seismic intensity, crater and active faults, and a suitability evaluation conducted for sedimentary basins of CO_2_ geological storage in China (CAGS1 internal report, 2012)^78^. The present report divides the evaluation results of suitability into five categories: not suitable, low suitability, normal suitability, high suitability, and very suitable. Most inland sedimentary basins in China are suitable for CO_2_ geological storage. Areas with active faults and volcanoes such as part of the minority region in the northwestern Ordos and southern Songliao basins are not suitable for CO_2_ geological storage. Basic data of environmental elements include GIS data of surface water, centralized drinking water source protection areas, nature reserves, national forest parks, national parks of China, and national key ecological function areas. Basic data were organized in the Geodatabase during spatial analysis and pre-processing. The spatial resolution of data was uniformly set to 1 km to facilitate analysis with the spatial model. The environmental suitability of a grid unit was determined as the minimum rating value of the indices. The specific data are shown in Table 3 and Fig. 3.

### Data availability statement

The data used in this study may be limitedly or full accessed by contacting the corresponding author.

## Results and Discussion

### Spatial patterns

The suitability results of environmental optimization of site selection for CCS in China can be obtained based on the analysis and evaluation of the spatial model employed (Fig. 4). Four categories of suitability classes were developed, i.e. classes I, II, III, and IV. Areas identified as suitability class I are prohibited regions, i.e. regions that are completely unsuitable for carrying out CCS projects. The suitability of classes II, III, and IV indicates that the environmental suitability of a CCS site selection has successively increased. From an environmental perspective, suitability class IV regions are relatively ideal areas for CCS projects. Most regions with a high environmental suitability (classes III and IV) in China are located in western China, and are concentrated in Xinjiang Uyghur Autonomous Region (Xinjiang; Fig. 4). The CCS site selection in eastern China may be greatly affected by the indices of population distribution and population activity intensity. In China’s western region, the CCS site selection may be affected by indices such as the ecological function zoning. Because prohibited areas for CCS sites such as water bodies, national forest parks, and nature reserves are relatively dispersed in China, the CCS site selection should reasonably avoid these areas after field surveys are completed.

Several basins provide ideal regions for CO_2_ geological storage with good environmental suitability including the central Tarim, northern Qaidam, northern Junggar, and the central Turpan-Hami basins as well as the northern margins of the Ordos and Erlian basins, and the western margin of the Hailar Basin. In particular, the central Tarim, the northern Qaidam, and the northern Junggar basins have larger areas available for CCS with strong environmental suitability. The primary trap of a structural unit in the Tarim Basin has a large potential for geological storage of CO_2_^64, 79, 80^, and the region is mainly located in the Kuqa depression, north depression and the central uplift area. These three target areas may be treated as the main site of CCS projects in the future as because they are also close to the oil and gas reservoirs in the Tarim Basin.

Less suitable sites for CCS in the western margin of the Tarim and Erlian basins, the central Hailar Basin, and the central Ordos Basin based on the environmental constraints of the main function zones. These include areas such as the Tarim River desertification control and ecological area, Yinshan mountain grassland, the Hulunbuir grassland-meadow, and the water and soil conservation areas of the Loess Plateau. Areas that provide only very limited suitability for CCS in the southern Junggar Basin because they are restricted by national ecological function zoning include important regions of water and biodiversity conservation in the Tianshan, and important regions of biodiversity conservation and sand-fixing in the western and eastern Junggar Basin.

### Characteristics of suitable regions

**Figure 5:**
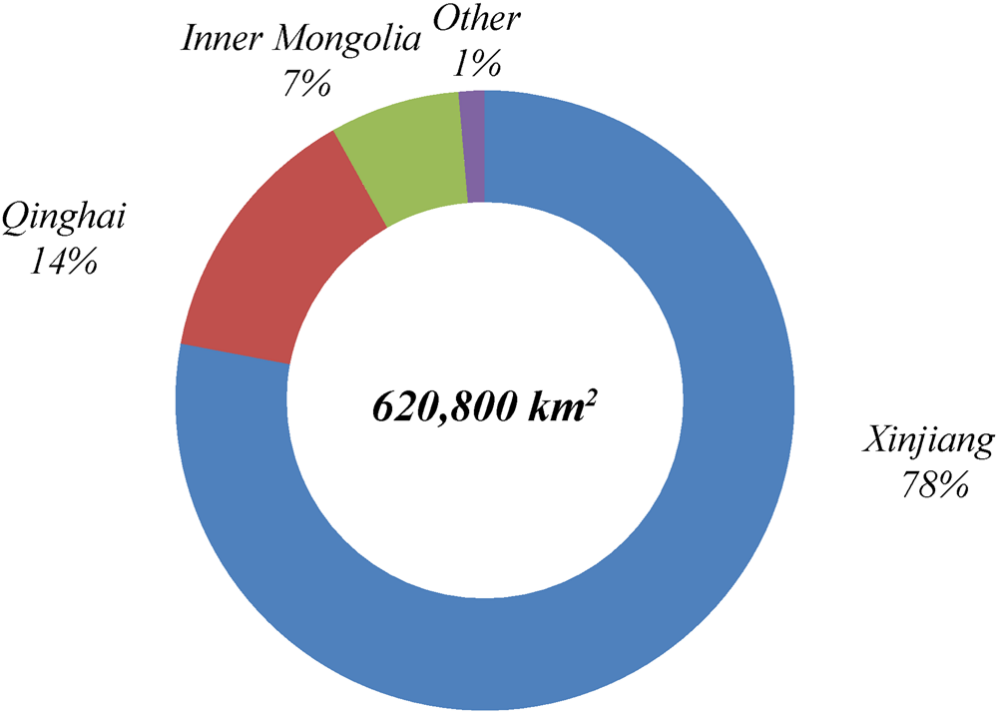
Area and proportion of environmental suitability for Class IV CO_2_ capture and storage sites in China.

The areas that are most suitable for CCS (class IV) in China cover a total area of 620,800 km^2^ (Table 4) and most of these areas are located in Xinjiang. Xinjiang includes 483,700 km^2^ of suitability class IV, accounting for 78% of the total area of suitability class IV. Qinghai Province includes an area of 86,400 km^2^ of suitability class IV, or 14% of the total area in class IV. Inner Mongolia Autonomous Region (Inner Mongolia) covers 42,200 km^2^ of suitability class IV, with 7% of the total. The regions in suitability class IV in Xinjiang, Qinghai and Inner Mongolia account for 99% of the country’s total area in this class (Fig. 5).

**Table 4:** Characteristics analysis of environmental suitability regions of China’s CCS site selection.

Suitability division		Class			
		I	II	III	IV
Area (km^2^)		645,206	586,654	156,616	620,828
NPP (10^−4^ kg C/m^2^)	Min	0	0	0	0
	Mean	4,046	2,674	1,791	1,338
	Max	15,971	15,123	4,663	2,339
Population density (person/km^2^)	Min	0	0	0	0
	Mean	335	28	6	0
	Max	68,693	22,161	4,896	4,454
Human activity intensity	Min	0	0	0	0
	Mean	6	0	0	0
	Max	11,779	5	1	0

**Figure 6:**
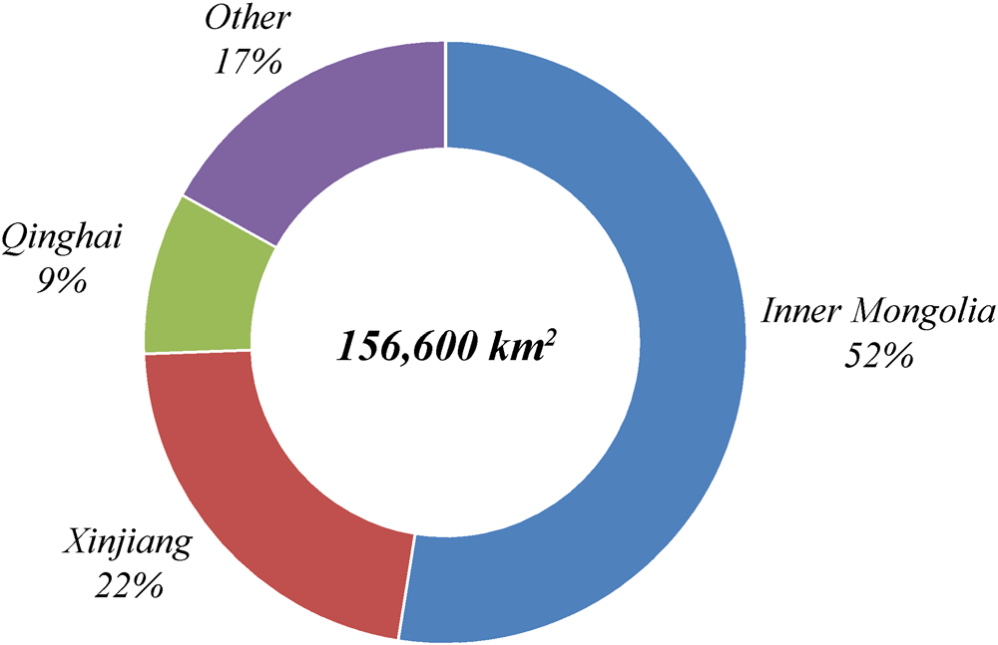
Area and proportion of environmental suitability for Class III CO_2_ capture and storage sites in China.

The areas of environmental suitability class III cover 156,600 km^2^ (Fig. 6), with most of this area lying in Inner Mongolia which has 82,200 km^2^ and 52% of the total area in suitability class III. Xinjiang has 34,300 km^2^ (22%) in class III while Qinghai Province has 13,660 km^2^ (9%). The combined area of Inner Mongolia, Xinjiang, and Qinghai in class III accounts for 83% of the total area of suitability class III.

From an environmental perspective, the regions of suitability class III and IV will be the ideal regions for CCS projects. Therefore, from a macro perspective, the Xinjiang, Qinghai, and Inner Mongolia region, usually called the Big Three region as one collective environment-friendly region for CCS in China, are regarded as the first priority region for strategic deployment of China’s CCS projects. In particular, the Big Three region is well aligned with the location of China’s coal chemical industry planning during the past decade^81, 82^ and early planning for CO_2_ enhanced water recovery technology announced during the China-U.S. Joint Announcement on Climate Change and Clean Energy Cooperation released on November 11, 2014^83, 84^.

**Figure 7:**
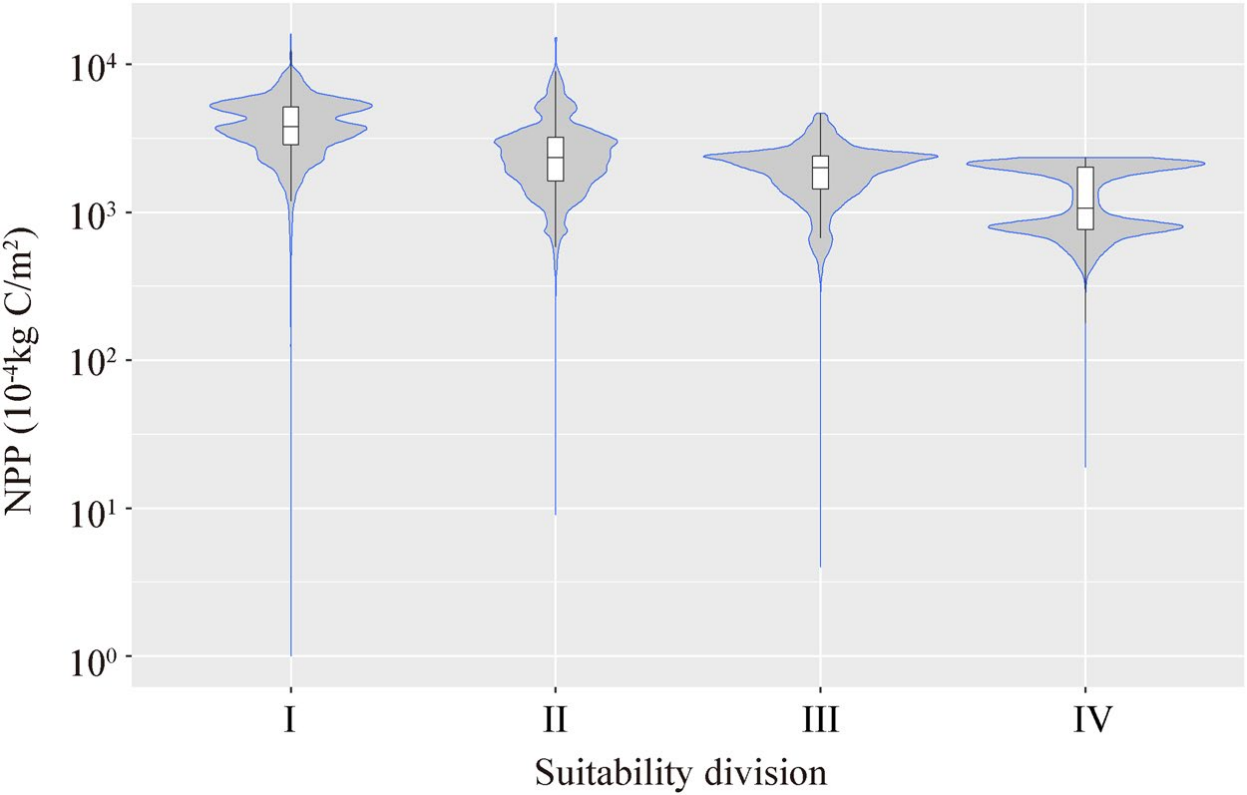
Distribution characteristics of net primary production in different regions of environmental suitability classes related to CO_2_ capture and storage. Note: The violin plots show the probability density of the data at different values on each side. The bottom, the band inside and top of the box plot inside the violin plot indicate the first quartile, the median and fourth quartiles of the data.

**Figure 8:**
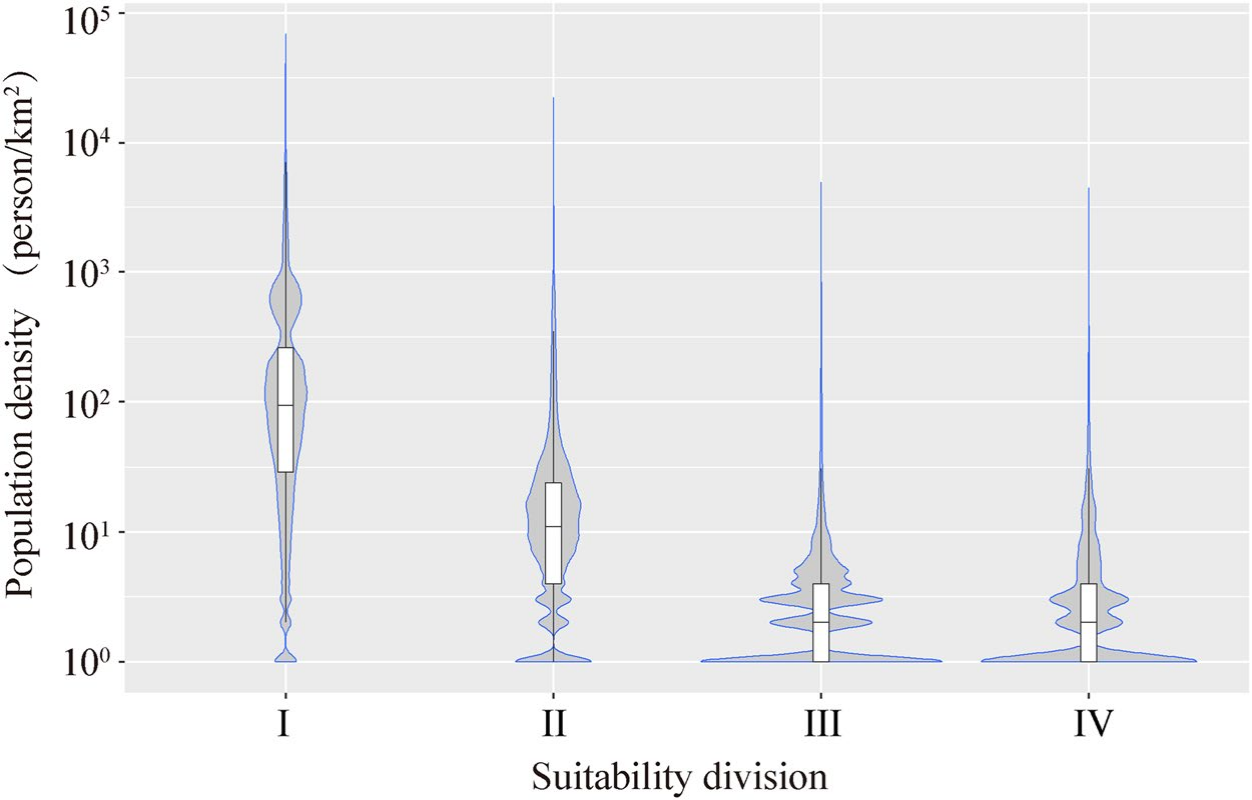
Distribution characteristics of the human population under different regions of environmental suitability classes related to CO_2_ capture and storage. Note: The violin plots show the probability density of the data at different values on each side. The bottom, the band inside and top of the box plot inside the violin plot indicate the first quartile, the median and the fourth quartiles of the data.

Vegetation net primary production (NPP), population density and the intensity of the activity of the population are important quantitative indices used during the evaluation of environmental suitability for CCS site selection. Based on the statistical characteristics of various environmental elements of evaluated regions within different classes of suitability (Figs 7 and 8), the analysis results show that regions with relative high environmental suitability (classes III and IV) have relatively low NPP and population density. Regions with the lowest environment suitability (class I) exhibit the highest values for both the maximum and median values of NPP and population density. As for environmental suitability class IV, the statistical distribution of NPP values shows a significant bimodal phenomenon, indicating that this class of region may have two different types of vegetation or quite different land cover types. However, generally speaking, class IV regions have relatively lower NPP compare to other regions.

The probability density curves of the population density have shown multiple peaks in the four classes of environmental suitability regions (Fig. 8). This statistical characteristic indicates that the regions for environmental suitability can still be divided further into sub-regions based on population density or urbanization and economic development, and knowing this will provide more sophisticated guidance for the spatial layout and site selection of CCS projects.

### Regional screening

Prefecture-level regions constitute the second level of the administrative structure in China, ranking below provinces and above counties. Considering the relatively manageable area and more powerful governing capacity by means of regulations and standards than counties, prefecture-level regions are regarded to be more fundamental and flexible in carbon mitigation and policy implementation compared to provinces and counties. Considering the region of environmental suitability class IV is the first priority for the selection of sites for CCS projects, this section is focused on the distribution of environmental suitability class IV in Chinese prefecture-level regions.

The spatial extent of environmental suitability class IV is far less than 500 km^2^ in the majority of Chinese prefecture-level regions. These areas are spatially fragmented and are not favorable as locations for CO_2_ geological storage projects. Three sedimentary basins, i.e. Tarim, Junggar, and Turpan-Hami, in Xinjiang have rich oil and gas resources. The geological conditions and geological processes for hydrocarbon accumulations in these three basins determine whether or not the reservoirs have good geological traps and reservoir-caprock combinations. Meanwhile, environmental elements which constrain the deployment of CCS projects are relatively fewer. Therefore, Xinjiang has the largest area with good environmental suitability for sites of CCS projects. In Xinjiang, three prefecture-level regions including Bayingol Mongolian Autonomous, Hotan, and Aksu prefectures not only have a large area with a continuous distribution for potential CCS sites with environmental suitability class IV, this region also has a high proportion (56%) of the total class IV area in China (Fig. 9).

Western Hulunbuir of Inner Mongolia has single area of 9,900 km^2^ of environmental suitability class IV for CCS site selection while eastern Xilingol League has about 26,400 km^2^. Meanwhile, northern Ordos has about 5,200 km^2^ and Qinghai Haixi Mongolian-Tibetan Autonomous Prefecture has about 86,300 km^2^. A single area ranked as class IV in eastern Jiuquan City covers about 4,400 km^2^, while the class IV area in Yulin and Yan’an in northern Shaanxi Province is more dispersed, with a total area of 145 km^2^.

### Validation for the target area of the Yanchang CCUS project

This section analyzes the appropriateness of environmental suitability division for China’s CCS site selection by using the pilot site, the Yanchang CCUS project in the Ordos Basin, China, as a case study. The full name of the Yanchang CCUS project is the Shaanxi Yanchang Petroleum CO_2_ capture, utilization and storage project. The Chinese scientists and government regulators believes this CO_2_ enhanced oil recovery (CO_2_-EOR) demonstration project is very important. In 2015, the Yanchang Petroleum Jingbian CCUS project passed international certification of the Carbon Sequestration Leadership Forum (CSLF), which became the first independently certified CCS project in China. On 25^th^ September 2015, the *U*.*S*.*-China Joint Presidential Statement on Climate Change* made it clear that the CCUS project mentioned in the *U*.*S*.*-China Joint Announcement on Climate Change* in 2014 will be determined in Yanchang oilfield. In addition, the potential site will be selected in the Yan’an-Yulin area. The Yanchang Petroleum Jingbian CCUS project is located at Qiaojiawa Village, Xiaohe Township, Jingbian County, Shaanxi Province^85^. The project began in 1st January 2012, and the deadline for releasing the results of the first phase of the research was 30^th^ April 2015. CO_2_ injection started on Sept. 4^th^, 2012 with twenty-ton tanker trucks used to transport CO_2_ during the first phase. The CO_2_ sources of the Yanchang Petroleum Jingbian CCUS project were purchased from the Xingping fertilizer plant in the western part of Xi’an before the Yulin coal chemical facility with CO_2_ capture ability of 50,000 tons per year was put into production by Yanchang Petroleum in 2012^86^. By the end of 2015, the total CO_2_ injection volume reached to 55,000 tons. This CCUS project is located in the Ordos Basin in the north central area of slope of northern Shaanxi, an area with stable regional tectonic conditions and free of large-scale tectonic activity and faults^85, 87^. Therefore, the geological structure of this region is stable and the occurrence of CO_2_ leakage caused by large scale tectonic activity or fracturing is relatively low. The Yanchang CCUS project is located in a region of suitability class III (Fig. 10) and the environmental sensitivity of this site is rather low. However, from the regional point of view, the environmental suitability class III of this region is fragmented in scope and water resources protection areas exist in the northwest. Therefore, the next phase of CO_2_ injection and other activities in this region need to fully consider the nearby environmental elements and conducting an environmental risk assessment is necessary.

## Conclusions

As one of the important mitigation measures for global greenhouse gas emissions, CCS technology is attracting a great amount of attention and has been developing rapidly in China. However, environmental risks and impacts of CCS projects have not yet been fully considered by the government and members of the public in China. By analyzing the on-the-ground situation and characteristics of China’s environmental management techniques, this paper uses the ecological red line as a reference, and analyzed the environmental suitability of CCS projects in China based on big data related to basic environmental issues in China. The results showed that regions of suitability classes III and IV, with very good environmental suitability for CCS projects, cover about 620,800 and 156,600 km^2^, respectively, and are mainly in three provinces in China, i.e. Xinjiang Uyghur and Inner Mongolia autonomous regions and Qinghai Province. In these three regions, the area of suitability class IV, the highest environmental suitability class not only accounts for a large land area, but also forms a continuous unit. These include in Bayingol Mongolian Autonomous Prefecture, Hotan Prefecture, Aksu Prefecture, Hulunbuir, Xilingol League and other prefecture-level regions. This large area may favor the deployment and implementation of CCS projects. In China, the current CCS projects are mainly considered CO_2_ emission sources and entail economic costs, e.g. Shenhua CCS and Yanchang CCUS. These have not placed adequate emphasis on environmental concerns. Along with the accumulation of experience and technical progress of China’s CCS pilot and demonstration projects, China is gradually considering the planning and deployment of CCS strategically at the national level. This study provides timely and strong support of the spatial layout and environmentally-sound management of CCS projects for decision-makers.

1. Xinjiang, Qinghai and Inner Mongolia and other western provinces have large areas with optimal environmental suitability for CCS site selection and projects where would have relatively little environmental impact. Furthermore, these regions are currently experiencing a large amount of oil and gas exploration as well as support coal and chemical-related industries. Therefore, the strategic deployment of a national level CCS can prioritize these regions.
2. The region with the highest potential for acceptable environment suitability for CCS site selection in China is mainly located in the west. However, the largest emission sources in China mainly occur in the east. Environmentally suitable areas for CCS sites do not spatially match with large CO_2_ emission sources, which create considerable difficulty for CCS planning. On this point, the establishment of a national CCS management network may resolve the problem to a certain degree in the future. Considering transportation costs of CCS projects, although some eastern regions that are close to large CO_2_ emission sources are less suitable for CSS, these areas should be given our full attention during the careful selection of potential CCS sites. It is appropriate to choose ocean-based geological storage if no on-shore sites are available.
3. The process of environmental analysis and management during the CCS site selection needs to be strengthened further. The present study was conducted on the national level. The strategic layout for CCS projects in different regions should not only consider the emission sources and economic costs, but should also fully account for environmental issues. In addition, the aforementioned analysis shows that the environmental issues still have large ramifications even for regions with the same level of suitability. Therefore, the refinement of spatial environmental management and further analysis are also crucial.

The present study started by referring to the standard technology and methods provided in China’s ecological red line assessment, which minimizes the need for subjective analysis and assessment. Next, after collecting and analyzing vast amounts of environmental data in as much detail as possible, we carried out model calculations on geographical units with a high spatial resolution. However, future work should still provide some improvements on the baseline established here as follows. (a) Regional differences of environmental elements are considered inadequate for spatial modeling on a national scale. Therefore, our research group plans to consider more precise assessments for each sub-region as the next step. (b) The accuracy of some of the data needs to be improved. For example, some nature reserves were only considered based on the location of the center of reserves and the land areas covered. Therefore, the actual spatial boundaries had to be substituted by a circle in the model used here. (c) It is very necessary to consider source-sink matching characteristics at a regional level, so as to further refine the sequestration potential for regions that are environmentally suitable for CCS projects, and to enhance the analysis of the integrated CCUS system. (d) The input data should be further refined to the level of municipal administrative units. It is crucial to provide a reference of CCUS site selection and make this available for local environmental protection departments.
